# Photophysical Properties of Nitrated and Halogenated Phosphorus Tritolylcorrole Complexes: Insights from Theory

**DOI:** 10.3390/molecules23112779

**Published:** 2018-10-26

**Authors:** Marta Erminia Alberto, Bruna Clara De Simone, Gloria Mazzone, Nino Russo, Marirosa Toscano

**Affiliations:** Dipartimento di Chimica e Tecnologie Chimiche, Università della Calabria, Cubo 14C, Via P. Bucci, 87036 Arcavacata di Rende, CS, Italy; malberto@unical.it (M.E.A.); gmazzone@unical.it (G.M.); m.toscano@unical.it (M.T.)

**Keywords:** phosphorus tritolylcorrole, photodynamic therapy, density functional theory, spin-orbit coupling, heavy atom effect

## Abstract

The photophysical properties of a series of nitrated and halogenated phosphorus tritolylcorrole complexes were studied in dichloromethane solvent by using the density functional theory. Particular emphasis was given to the absorption spectra, the energy gap between the excited singlet and triplet states, and the magnitude of the spin-orbit couplings for a series of possible intersystem crossing channels between those excited states. The proposed study provides a better description of the photophysical properties of these systems while giving insights into their possible use as photosensitizers in photodynamic therapy.

## 1. Introduction

Starting from the first reported synthesis of corroles by Johnson and Kay in 1964 [1], the interest in these porphyrin-like systems has grown considerably due to advances in their efficient synthesis combined with relatively easy options for varying the axial ligands, the central metal atom and the peripheral substituents. Their distinctive properties, often different from the analogues porphyrin systems, allow many applications in different fields of modern chemistry [2,3]. In particular, free ligand corroles and their metal coordinated derivatives have been proposed as applicable to optical imaging, dye-sensitized solar cells [4], electron and energy transfer systems [5], photodynamic detection [6], drug candidates against oxidative stress [7], catalysis [8] and photodynamic therapy [9]. Research has mainly focused on bare corroles and their complexes with different metal ions [2,3,10,11]. Also, corrole complexes with nonmetals have received considerable interest in recent years [12] and the synthetic strategies and characterization of the P(V) complexes have opened a new horizon in corroles chemistry [13,14,15,16,17]. Amongst these, halogenated derivatives have attracted particular interest due to the presence of halogen atoms, which provide very interesting photophysical properties to these systems [13,14,15,18]. Pomarico et al. [13] have recently synthesized a series of phosphorus (V) complexes of 4,10,15-tritolylcorrole in which -NO_2_ and -Br groups are present in different amounts and peripheral positions. In order to better elucidate the photophysical properties of these systems, we have undertaken a careful theoretical study on some nitrated and halogenated phosphorus tritolylcorrole complexes by using the density functional level of theory. Particular emphasis has been devoted to those photophysical parameters that are crucial for the utilization of these systems as photosensitizers in photodynamic therapy. Moreover, since an efficient intersystem crossing is an essential requirement for a photodynamic therapy (PDT) candidate, to improve those physical properties we also introduced a structural modification in two of the investigated compounds by replacing the bromine with an iodine substituent and analyzing the resultant effect.

PDT is a mini-invasive procedure used in different medical applications ranging from tumor therapy, atherosclerosis, and inactivation of some bacteria and viruses and insecticides [19,20,21,22]. The key cytotoxic agent in PDT is the singlet molecular oxygen (^1^Δ_g_) generated in situ by energy transfer from an excited sensitizer to the ground electronic state of oxygen molecules (^3^Σ_g_). Consequently, despite its success [23,24,25,26,27], one of the main drawbacks of PDT is associated with the lack of oxygen usually observed in several tumors. Indeed, in hypoxic conditions, where oxygen concentrations are already low due to slow diffusion of the gas molecule in the tumor tissue, PDT may fail, leading to resistances [24,27], and other approaches should be used [28]. On the contrary, in oxygenated environments, the therapy can be applied and usually starts with the administration of a photosensitizing agent (PS) which is excited from its electronic ground state (S_0_) to a singlet excited state (S_n_) by using light of a specific wavelength and intensity. The excited triplet state generated through a radiation-less intersystem crossing transition, under certain conditions can transfer its energy to the ground state molecular oxygen (^3^Σ_g_) generating cytotoxic singlet oxygen (^1^Δ_g_) (type II photoreactions). The excited PS can also react directly with the organic substrates present in the irradiated tissue by electron exchange, producing radical intermediates that are subsequently scavenged by oxygen, with the formation of the superoxide oxygen radical species and other highly reactive radicals (type I photoreactions). 

In order to obtain a type II reaction working mechanism, an efficient PDT photosensitizer should possess the following main photophysical properties:

(a) a low fluorescence quantum yield, which ensures a higher probability that the excited state S_1_ could decay into the triplet excited one;

(b) a triplet state with sufficient energy to generate the excited ^1^∆_g_ molecular oxygen. Since the energy necessary to obtain excited molecular oxygen is known to be 0.98 eV, [29] the PS triplet excited state must lie above this limit;

(c) an efficient intersystem crossing between singlet and triplet states. To do this, a high spin–orbit coupling constant is necessary as it can be deduced from the expression of the ISC kinetic constant derived from the Fermi Golden Rule [30].

Furthermore, in order to maximize the tissue penetration, an appropriate absorption wavelength falling within the so-called therapeutic window (550–850 nm) is required.

Of course, other particular properties such as solubility in aqueous media, redox stability, absence of intermolecular aggregation phenomena which decreases the photodynamic action, and no toxicity in the dark, are necessary to propose new molecules as PDT photosensitizers [31].

## 2. Computation Details

All the calculations were performed at the density functional theory level (DFT) and its time-dependent density functional linear response formulation (TDDFT) as implemented in the Gaussian09 code [32]. No symmetry constraints were imposed during the geometry optimizations. On the basis of our previous experience of the photophysical properties of similar systems [33,34,35,36], ground and excited state optimization were performed by using the B3LYP [37,38] exchange and correlation functional coupled with the 6-31G* basis sets for all the atoms excepts for iodine for which the SDD pseudopotential [39] was employed. 

The lowest twenty vertical excitation energies were calculated on the previously optimized geometries by adding a diffuse function to the basis set (6-31 + G*). Solvent effects were included by using the non-equilibrium implementation [40] of the polarizable continuum model [41], taking into account the dielectric media of dichloromethane (ε = 8.93) since the experimental data are available for that solvent.

Spin-orbit matrix elements $\langle S_{1}|H_{SO}|T_{j}\rangle$ were computed by using the quadratic-response TDDFT approach [42,43], as implemented in the Dalton code [44]. For compounds **4** and **5** where iodine atoms are present, the spin–orbit coupling operators for effective core potentials with an effective nuclear charge [45] was employed, as also previously done with systems containing heavy atoms [46,47,48]. The atomic-mean field approximation [49] was used instead for the other cases. For this purpose, B3LYP functional in conjunction with cc-pVDZ basis set was employed for all the atoms except iodine, for which the coupled pseudopotential was considered. 

## 3. Results and Discussion

**Scheme 1:**
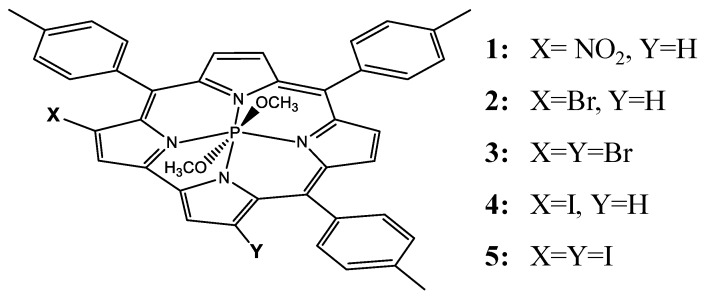
Schematic representation of compounds investigated in this work.

The structures of the five examined systems are depicted in Scheme 1. 

The phosphorus atom is always coordinated by two -OCH_3_ group in the axial position and through the four nitrogen atoms of the corrole ring. The presence of different peripheral substituents (X and Y in Scheme 1) in compounds **1**–**5** allow us to shed light on the effect of a mono- or bi-heavy atom substitutions on these kinds of systems.

The computed vertical excitation energies for singlet and triplet states, their oscillator strengths, the nature of the orbitals involved in each transition together with the available experimental information, are collected in Table 1. 

Comparison with the experimental data, which is only possible for compounds **1**–**3** in dichloromethane [13], reveals an overall satisfactory agreement between the experimental and computed spectra. The average error registered is 24 nm and the maximum deviation (44 nm) occurs for the S_1_ absorption of compound **1**.

**Figure 1:**
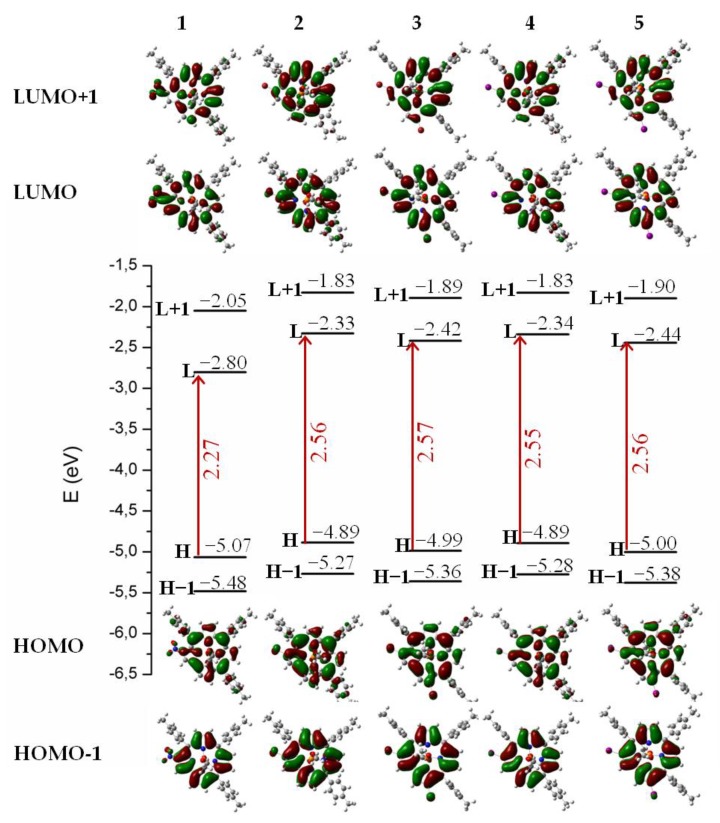
Energy diagram of the four Gouterman’s orbital and their counter plot for the ground states of compounds **1**–**5**. H-L energy gaps (eV) are indicated by solid red arrows.

The substitution of the NO_2_ group with a halogen atom causes a significant blue shift of the maximum absorption S_1_ band for all the investigated compounds **2**–**5,** When a bromine or an iodine replace the nitro group in structures **2** and **4**, the S_1_ band experiences a shift of 114 nm toward smaller wavelengths, and the computed spectra of the mono-halogen substituted corroles **2** and **4** are essentially the same. The introduction of the second halogen atom in Y position, generating structures **3** and **5**, does not introduce any significant effect on the UV-VIS spectra compared to **2** and **4**, as can be deduced from Table 1. Actually, the nitro group significantly stabilizes the LUMO (L) orbital with a consequent reduction in the H-L gap compared with those observed for the halogen-derivatives. Indeed, looking at the Gouterman frontier orbital energies (Figure 1), the H-L energy gap goes from the value of 2.27 eV in **1** to about 2.56 eV in the other compounds. 

Two triplet states, T_1_ and T_2_, and another three (T_1_, T_2_ and T_3_) lie below the first S_1_ state, for **1** and for **2**–**5** compounds, respectively, as can be observed in Table 1. All the triplet states possess sufficient energy to promote the formation of the cytotoxic excited singlet oxygen species ^1^Δ_g_, that is, their energy is higher than the 0.98 eV required for the O_2_ triplet to singlet transition. The T_1_ states are all originated by H→L transitions while T_2_ is mainly (92%) H − 1→L in nature in complex **1**. The situation is different for the halogenated systems in which T_2_ and T_3_ are always originated from H→L + 1 and H − 1→L transitions with almost the same contribution (about 50%), as reported in Table 1. The presence of peripheral halogen substitution increases the T_1_ energy value as a result of a destabilization of the LUMO orbital that is observed when the nitro group is replaced by a halogen substituent. Indeed, the T_1_ energy passes from 1.18 eV in the case of molecule **1** to about 1.40 eV in the **2**–**5** complexes.

As discussed in the introduction, besides the need for the correct energy value of the triplet states, a photosensitizer should possess a low fluorescence quantum yield and an efficient nonradiative intersystem crossing between singlet and triplet electronic states to produce the singlet oxygen molecule.

Generally, the presence of halogen or other heavy atoms decreases the fluorescence decay and increases the ISC rate constant in different kinds of molecular systems [47,48,50,51]. This effect has been also observed in corroles complexes. In particular, Shi et al. [52], have shown as the presence of one iodine on the aryl ring of triarylcorroles induces a 4-fold decrease in fluorescence quantum yield and a 60-fold increase in intersystem crossing rate constants. More recently, similar behaviors have been observed in other iodinated corrole complexes [18]. Since the ISC rate constant depends on the magnitude of the spin-orbit coupling, we have calculated the $\left|\langle \Psi_{S_{1}}|{\hat{H}}_{so}|\Psi_{T_{n}}\rangle \right|$ values for the different S_1_–T_n_ possible ISC channels. The obtained results are collected in Table 2 together with the S_1_–T_n_ energy gaps. 

From the values reported in that table, it is evident that the effect of the peripheral bromine substitution in compounds **2** and **3**, is negligible for all the considered spin-orbit coupling (SOC) constants.

On the contrary, iodine causes SOCs to increase by one or two order of magnitude compared with brominated ones and the S_1_–T_2_ channel to have the highest value.

**Figure 2:**
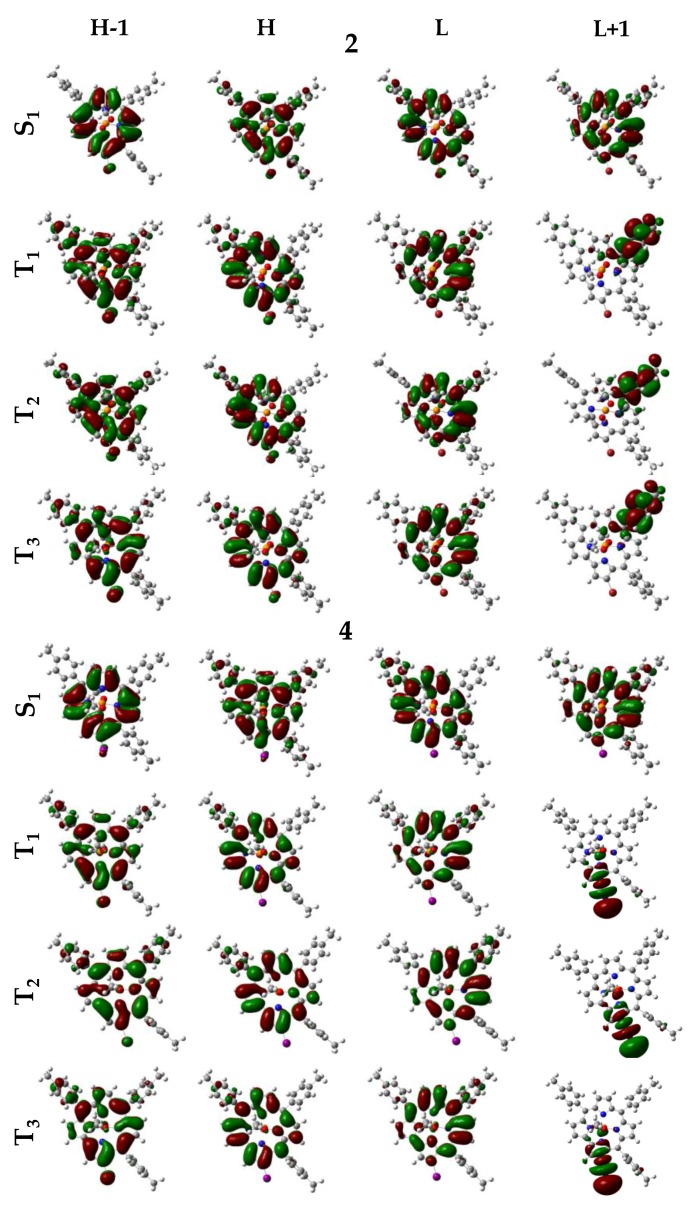
Molecular orbital contour plots for selected excited states of systems **2** and **4**.

The Gouterman molecular orbital plots for the excited singlet and triplet electronic states involved in the considered ISC events for system **2** and **4** are reported in Figure 2, to provide some insights on the origin of these effects. Actually, the matrix elements of the spin-orbit couplings vary strongly from compound **2,** containing the bromine atom to **4**, in which an iodine is present instead. Thus we chose to compare these two systems.

According to the El Sayed rules [53], high SOC values are obtained if the transition takes place with a change in the composition of the molecular orbitals involved. Therefore, we analysed the most important channels for compounds **2** and **4.**

The molecular orbitals involved in the ISC between S_1_ and T_1_ in compound **2** and **4** are HOMO and LUMO (Table 1), and both possess a π character (Figure 2). Due to the similar nature of the involved orbitals, the increment of SOC values due to the presence of a heavy atom like iodine in **4** is not so significant for this channel. On the contrary, the computed values for S_1_–T_2_ ISC mechanisms reveal an important role of the iodine substituent. Actually, the S_1_–T_2_ ISC for compound **2** occurs between a π-π* state (S_1_, H→L) to another π-π* state (T_2_, H→L + 1), as it can be deduced looking at Figure 2. When iodinated compound **4** is considered, the nature of the molecular orbitals involved in the ISC channel change significantly. Indeed, while the S_1_ state is still characterized by a H→L transition having both a π nature as in the case of molecule **2**, the T_2_ state is different since the L + 1 is now entirely located on the lone pairs of the iodine atom. This change in symmetry, as predicted by the El Sayed rules, causes a significant increase of the SOC value for the S_1_–T_2_ coupling.

A less important increase in the SOC value was obtained for the S_1_–T_3_ channel of compound **4** compared to molecule **2**. Indeed, different to what was observed in the previous case, the contribution of the H→L + 1 transition is no more dominant in T_3_, being the latter one characterized mainly by a H − 1→L, π-π* transition. This is why the increment in the SOC value for compound **4** is not as big as that observed for the S_1_–T_2_ channel. 

Looking at the obtained values of the spin−orbit coupling for the considered ISC processes, in principle, more than one deactivation pathways could be hypothesized. Starting from the low-lying populated S_1_ state (see the oscillator strengths in Table 1) and considering the presence of three triplet excited states below it for the halogenated systems, the following paths should be possible upon irradiation:

(a) $S_{0}\overset{A}{\to }S_{1}\overset{\mathrm{ISC}}{\to }T_{3}\overset{\mathrm{IC}}{\to }T_{2}\overset{\mathrm{IC}}{\to }T_{1}$
(b) $S_{0}\overset{A}{\to }S_{1}\overset{\mathrm{ISC}}{\to }T_{2}\overset{\mathrm{IC}}{\to }T_{1}$
(c) $S_{0}\overset{A}{\to }S_{1}\overset{\mathrm{ISC}}{\to }T_{1}$

Following the Fermi Golden Rule [30], the intersystem crossing kinetics are directly related to the spin–orbit matrix elements and the Franck−Condon weighted density of states (FCWD). In the framework of Marcus−Levich−Jortner theory [54], the FCWD is proportional to the difference between the energies of the singlet and the triplet states ΔE at their equilibrium geometry. Looking at Table 2, where both the computed SOCs and ∆Es are reported, path (b) is indicated as the preferred one due to the high value of spin−orbit matrix elements and a feasible ΔE between S_1_ and T_2_. A more precise prediction can only be made by calculating the kinetic constants. Nevertheless, this kind of parameter implies the calculation of the Franck-Condon weighted density of states which is highly expensive for the considered systems. Work is in progress in this area.

## 4. Conclusions

Density functional theory was employed to investigate a series of nitrated and halogenated phosphorus tritolylcorrole complexes focusing on some crucial photophysical properties required by a photosensitizer to be utilized in photodynamic therapy applications. The following conclusions can be made.

The presence of peripheral halogens in place of the -NO_2_ group, determines a significant blue-shift of the maximum near infrared absorption band. However, the absorption Q band of all the examined compounds is intense and with a wavelength that falls in the so-called photodynamic window.

For all the investigated compounds, the energy of the low-lying triplet state (T_1_) is sufficiently higher than the energy required to excite the molecular oxygen in its ^1^Δ_g_ state. 

The spin-matrix elements significantly increase when iodine atoms are present as peripheral substituents. For all the five examined compounds the computed SOCs are higher than the corresponding value for the Foscan^®^ [55] system already used in PDT.

We hope that our data could stimulate further investigations on iodine containing phosphorus tritolylcorrole complexes for their possible use as photosensitizers in photodynamic therapy.

## Figures and Tables

**Table 1:** B3LYP/6-31 + G * vertical excitation energies, ΔE (eV), λ (nm), oscillator strengths *f* and main transitions (H = HOMO, L = LUMO) for studied compounds, computed in dichloromethane. Experimental values are taken from [13].

Cmpd.	State	ΔE	λ	*f*	Transitions	λ_exp_
**1**	S_1_	1.84	673	0.275	H→L (93%)	635
	S_2_	2.19	566	0.161	H − 1→L (75%)	590
	S_3_	2.68	463	0.439	H→L + 1 (64%)	447
	S_4_	3.18	390	0.559	H − 1→L + 1 (29%), H − 4→L (29%)	428
	T_1_	1.18	1050		H→L (96%)	
	T_2_	1.62	766		H − 1→L (92%)	
**2**	S_1_	2.22	559	0.310	H→L (86%)	599
	S_2_	2.37	522	0.007	H − 1→L (54%), H→L + 1 (45%)	526
	S_3_	3.03	405	1.112	H→L + 1 (52%), H − 1→L (44%)	421
	S_4_	3.18	390	1.066	H − 1→L + 1 (82%)	410
	T_1_	1.41	879		H→L (99%)	
	T_2_	1.76	705		H→L + 1 (55%), H − 1→L (43%)	
	T_3_	1.96	632		H − 1→L (56%), H→L + 1 (43%)	
**3**	S_1_	2.24	555	0.295	H→L (86%)	599
	S_2_	2.39	520	0.011	H − 1→L (56%), H→L + 1 (43%)	527
	S_3_	3.07	404	1.176	H→L + 1 (54%), H − 1→L (41%)	422
	S_4_	3.21	386	0.995	H − 1→L + 1 (83%)	411
	T_1_	1.40	885		H→L (99%)	
	T_2_	1.78	697		H→L + 1 (50%), H − 1→L (48%)	
	T_3_	1.97	629		H − 1→L (51%), H→L + 1 (48%)	
**4**	S_1_	2.22	559	0.318	H→L, (86%)	
	S_2_	2.37	522	0.008	H − 1→L (54%), H→L + 1 (45%)	
	S_3_	3.04	407	1.101	H→L + 1 (51%), H − 1→L (42%)	
	S_4_	3.18	390	1.069	H − 1→L + 1 (82%)	
	T_1_	1.41	877		H→L (99%)	
	T_2_	1.76	706		H→L + 1 (54%), H − 1→L (44%)	
	T_3_	1.96	632		H − 1→L (56%),H→L + 1 (43%)	
**5**	S_1_	2.23	556	0.311	H→L (86%)	
	S_2_	2.39	519	0.014	H − 1→L (57%), H→L + 1 (42%)	
	S_3_	3.05	406	1.186	H→L + 1 (53%), H − 1→L (40%)	
	S_4_	3.22	385	1.018	H − 1→L + 1 (82%)	
	T_1_	1.40	887		H→L (99%)	
	T_2_	1.78	698		H − 1→L (50%), H→L + 1 (48%)	
	T_3_	1.97	629		H→L + 1 (50%), H − 1→L (49%)	

**Table 2:** Spin–orbit matrix elements (cm^−1^) and singlet-triplet energy gaps (eV) calculated at the B3LYP/cc-pVDZ level of theory.

Cmpd.	$\left|\langle \Psi_{S_{1}}|{\hat{H}}_{so}|\Psi_{T_{1}}\rangle \right|$	ΔE_S1–T1_	$\left|\langle \Psi_{S_{1}}|{\hat{H}}_{so}|\Psi_{T_{2}}\rangle \right|$	ΔE_S1–T2_	$\left|\langle \Psi_{S_{1}}|{\hat{H}}_{so}|\Psi_{T_{3}}\rangle \right|$	ΔE_S1–T3_
**1**	0.48	0.66	0.35	0.22		
**2**	0.28	0.81	0.67	0.46	0.32	0.26
**3**	0.26	0.84	0.76	0.46	0.27	0.27
**4**	13.10	0.81	35.0	0.46	5.4	0.26
**5**	8.54	0.83	77.27	0.45	12.65	0.26
